# Neurodiagnostics in Sports: Investigating the Athlete’s Brain to Augment Performance and Sport-Specific Skills

**DOI:** 10.3389/fnhum.2020.00133

**Published:** 2020-04-09

**Authors:** Oliver Seidel-Marzi, Patrick Ragert

**Affiliations:** ^1^Institute for General Kinesiology and Exercise Science, Faculty of Sport Science, University of Leipzig, Leipzig, Germany; ^2^Department of Neurology, Max Planck Institute for Human Cognitive and Brain Sciences, Leipzig, Germany

**Keywords:** neuroplasticity, fNIRS, non-invasive brain stimulation, performance enhancement, neurodiagnostic, athletes, neuromodulation

## Abstract

Enhancing performance levels of athletes during training and competition is a desired goal in sports. Quantifying training success is typically accompanied by performance diagnostics including the assessment of sports-relevant behavioral and physiological parameters. Even though optimal brain processing is a key factor for augmented motor performance and skill learning, neurodiagnostics is typically not implemented in performance diagnostics of athletes. We propose, that neurodiagnostics via non-invasive brain imaging techniques such as functional near-infrared spectroscopy (fNIRS) will offer novel perspectives to quantify training-induced neuroplasticity and its relation to motor behavior. A better understanding of such a brain-behavior relationship during the execution of sport-specific movements might help to guide training processes and to optimize training outcomes. Furthermore, targeted non-invasive brain stimulation such as transcranial direct current stimulation (tDCS) might help to further enhance training outcomes by modulating brain areas that show training-induced neuroplasticity. However, we strongly suggest that ethical aspects in the use of non-invasive brain stimulation during training and/or competition need to be addressed before neuromodulation can be considered as a performance enhancer in sports.

## Introduction

*“Citius, Altius, Fortius”—*Boosting motor performance and skills in athletes on a relatively short time scale and with little effort is a desired goal in professional sports. Athletes typically need to invest a lot of effort and strenuous successive practice over many years. As a rule of thumb, according to Ericsson et al. (1993), a minimum of 10 years or 10,000 h of intense practice is necessary to become an expert in a specific sports discipline. In almost all sports, performance diagnostics is a vital component for athletes to quantify individual performance levels, to evaluate training success and to guide training regimes. The standard procedure for such diagnostics comprises a combination of sports-related behavioral tasks and selected performance-relevant physiological parameters such as heart rate variability, lactate concentration or oxygen consumption.

However, neurodiagnostic tools to evaluate brain processing during sports-related movements are typically not implemented in performance diagnostics of athletes. This seems to be surprising since the central nervous system initiates voluntary movements by generating neural impulses that control the execution of movements. Furthermore, there is neuroscientific evidence that optimal brain processing is a key factor for enhanced motor performance or skill learning. So why is it that performance diagnostics in athletes is not routinely considering neurodiagnostic tools to assess such a brain-behavior relationship? A better understanding of such a relationship might help to optimize performance and/or learning capabilities in athletes. Although neurodiagnostics is an umbrella term for a huge variety of diagnostic tools, the present perspective paper focuses on the use of selected non-invasive brain imaging techniques for performance diagnostics in sport.

One possible explanation for the lack of use of neurodiagnostic tools in performance diagnostics is that over the past decades, the brain has not been considered as a performance inducing or enhancing determinant in sports. Hence, it is not surprising that only a little attention was paid to the role of optimal brain processing and its effects on motor performance or skill learning in athletes.

However, neurodiagnostic tools such as magnetic resonance imaging (MRI) or functional near-infrared spectroscopy (fNIRS) have been extensively used to assess brain-behavior relationships over the lifespan. Based on these investigations, we know that the brain adapts its function and structure according to environmental changes on a very short time scale (Lin et al., 2018; Burke and Barnes, 2006; Erickson et al., 2013; Smith, 2013; Pauwels et al., 2018). Apart from learning-induced functional brain adaptations, recent studies suggest that the effectiveness of movement control and motor skill learning also depends on the individual brain structure and its neuroplastic adaptation (Draganski et al., 2004; Taubert et al., 2010, 2011; Tomassini et al., 2011; Sampaio-Baptista et al., 2013). However, this accumulative evidence is primarily based on simplified models of movement and skill learning paradigms that do not necessarily reflect relevant neuroplastic adaptations in complex sports scenarios. Therefore, future studies should systematically quantify neural processing during the execution of sports-related movements in real-world settings.

In this perspective article, we discuss why and how neurodiagnostic tools should be implemented in diagnostic and training routines to shed more light on the role of optimal brain processing on performance levels in competitive sports. Furthermore, we argue that a better understanding of such a brain-behavior relationship and its training-induced adaptations might help to enhance performance levels and motor skill learning in athletes.

## Neurodiagnostics in Sports: Implications for Performance Enhancement in Athletes

### Diagnostics of Neuroplasticity Using Non-invasive Brain Imaging Techniques

**Figure 1:**
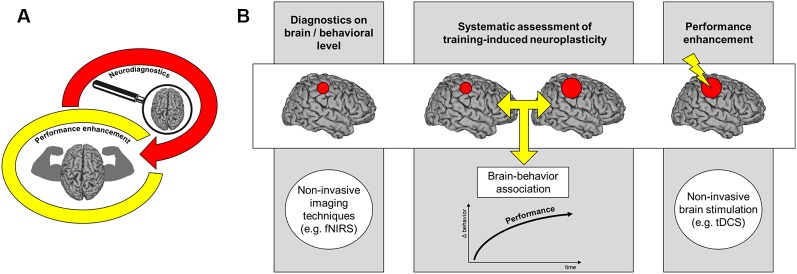
Neurodiagnostics in sports. The figures illustrate how neuroscientific methods might be integrated into behavioral diagnostics in athletes. **(A)** We propose that neurodiagnostics might help to augment performance levels in athletes. **(B)** Framework for performance enhancement in athletes using neurodiagnostic tools. Initially, diagnostics of functional and/or structural features of the brain and its relation to performance in sports is an important step towards performance enhancement in athletes. Characterizing training-induced brain changes might help to guide training processes and optimize training outcomes. Finally, targeted non-invasive brain stimulation such as transcranial direct current stimulation (tDCS) might help to enhance performance by modulating brain areas that show training-induced neuroplasticity.

Non-invasive brain imaging techniques are capable of quantifying expertise-related brain adaptations in various sports disciplines. For example, it has been shown that efficiency in sports is directly related to an optimized brain functioning. A recent functional MRI (fMRI) study by Naito and Hirose (2014) provided novel evidence that the brain of a top-class football player (Neymar) relies on less neuronal resources in motor-related areas as compared to other athletes. Similar findings have been found in other sports disciplines such as table tennis where athletes show less brain activation during the execution of sports-related and sports-unrelated visuospatial tasks as compared to non-athletes (Guo et al., 2017). Moreover, several studies provide compelling evidence that there is a causal relationship between brain activation and behavioral performance (Orban et al., 2010; Peterson and Fling, 2018) or motor skill learning capabilities (Sun et al., 2007; Wadden et al., 2013), respectively. Apart from functional alterations, regular training is also capable of inducing changes on a structural brain level. Meier et al. (2016) suggested that structural adaptations are even sport-specific and are manifested in brain regions which are essential for neural processing of sport-specific skills. They found an increased gray matter (GM) volume in the hand area of M1 of handball players as compared to non-athletes, whereas ballet dancers showed an increased GM volume in the foot area of M1. The aforementioned findings provide novel evidence that motor expertise is capable of modifying brain processing and morphology in a sports context. Furthermore, these findings implicate that the athlete’s brain seems to work more efficiently (Dunst et al., 2014) as compared to lower-level athletes and/or non-athletes. Similar findings have also been found in musicians (Strait et al., 2009; Medina and Barraza, 2019). However, since the aforementioned evidence about functional brain adaptations is solely based on performing simplified and mostly sport-unspecific movements, there is a lack of knowledge about brain processing and adaptations during the execution of sport-specific movements (see Figure 1A).

**Figure 2:**
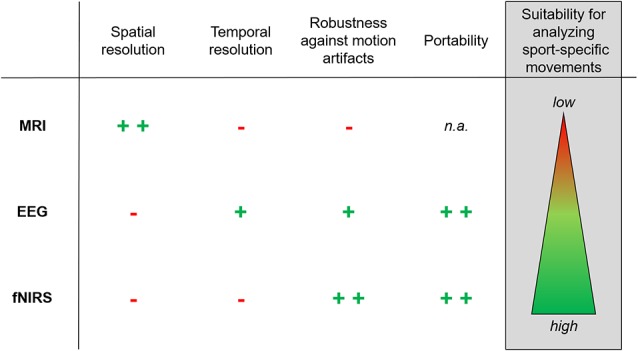
Recommendation for the use of non-invasive brain imaging techniques in neurodiagnostics of sport-specific movements. The figure illustrates advantages and disadvantages of magnetic resonance imaging (MRI), electroencephalography (EEG) and functional near-infrared spectroscopy (fNIRS) with regards to spatial resolution (i.e., *where* certain brain areas/networks are active), temporal resolution (i.e., *when* certain brain areas/networks are active), robustness against motion artifacts, portability of the neurodiagnostic tool and suitability for analyzing sport-specific movements (indicating implications for neurodiagnostics). Symbols are defined as follows: + +, very high; +, high; -, low; n.a., not applicable.

Beyond financial and infrastructural aspects, one crucial limitation in the use of MRI for neurodiagnostics is that sport-specific movements cannot be performed due to the spatial limitations inside the MRI bore and its high susceptibility to motion artifacts (Zaitsev et al., 2015; Havsteen et al., 2017; see Figure 2). Apart from these limitations in the use of MRI, it is reasonable to assume that a better understanding of brain functioning and/or adaptations in brain structure might help to optimize skills and performance in a sports-related context. Alternatively, a better characterization of the athlete’s brain might not only help to predict training success. Brain imaging techniques can additionally be used as a diagnostic tool to identify motor expertise and talent in various fields of sport.

One non-invasive brain imaging method that has been widely used in a sports-related context is electroencephalography (EEG). The major advantages of this method are on the one hand its portable application and on the other hand its high temporal resolution (i.e., important for research that aims to characterize when certain brain areas are active during movement execution) (van Gerven et al., 2009; Mehta and Parasuraman, 2013). More importantly, as compared to MRI, EEG provides a direct assessment of brain activity by recording voltage fluctuations at the head surface resulting from ionic current within the neurons of the brain (Light et al., 2010). Hence, EEG has been considered by numerous previous studies as a valuable tool to study neuronal activity during the execution of sports-related movements (Thompson et al., 2008; Park et al., 2015; Cheron et al., 2016). Particularly in sports disciplines characterized by a steady setting and minimal movements such as rifle shooting (Hillman et al., 2000; Doppelmayr et al., 2008), archery (Salazar et al., 1990; Landers et al., 1991) and golf (Babiloni et al., 2008), EEG has been successfully applied to investigate cortical activity during the execution of the respective sports discipline and its relation to the optimal performance or motor expertise. Furthermore, it has been shown that EEG is also feasible for more complex movements such as walking on a treadmill (Severens et al., 2012) and cycling (Brümmer et al., 2011; Ludyga et al., 2016). However, similar to fMRI, major disadvantages of EEG include its high susceptibility to motion artifacts (Symeonidou et al., 2018) and the relatively low spatial resolution (i.e., important for research that aims to characterize where certain brain areas are active during movement execution) as compared to fMRI (van Gerven et al., 2009; Mehta and Parasuraman, 2013; see Figure 2).

Thus, fNIRS is considered as a further promising non-invasive brain imaging method. Due to its portable application, fNIRS offers the opportunity to assess neuronal activity during the execution of sport-specific movements with a moderate to low (depending on inter-optode distance but typically in cm range) spatial resolution (van Gerven et al., 2009; Mehta and Parasuraman, 2013) and low susceptibility to movement artifacts (see Figure 2). fNIRS relies, in analogy to fMRI, on the principle of neurovascular coupling also known as the hemodynamic or blood oxygenation level-dependent (BOLD) response (Strangman et al., 2002; Liao et al., 2013). It involves the quantification of chromophore concentrations resolved from the measurement of relative changes in oxygenated (Hb) and deoxygenated hemoglobin (HHb) which are assumed to be indicators for changes in neural processing (Villringer and Chance, 1997; Obrig and Villringer, 2003). However, due to the time delay of hemodynamic response alterations, the temporal resolution of fNIRS recordings is less good as compared to fMRI and EEG (van Gerven et al., 2009; Mehta and Parasuraman, 2013; see Figure 2). Further limitations, as well as current contributions and possible prospects of fNIRS and EEG, are discussed in a recent position article by Perrey and Besson (2018).

Despite its limitations described above, previous studies have demonstrated that fNIRS is an appropriate and reliable method for measuring neural activity during simple and complex motor tasks (Leff et al., 2011; Pinti et al., 2018). Beyond that, portable fNIRS allows quantifying neural activity in real-world settings without movement constraints (Piper et al., 2014; Pinti et al., 2018). Hence, measuring brain activation during the execution of sports-related movements seems feasible. Many previous studies have successfully applied fNIRS to study functional brain adaptations during complex motor tasks such as juggling (Carius et al., 2016), balancing (Seidel et al., 2017), squatting (Kenville et al., 2017), climbing (Carius et al., 2020), playing table tennis (Balardin et al., 2017), running (Suzuki et al., 2004) and cycling (Seidel et al., 2019). Additionally, the focus is increasingly shifting in the direction of investigating neural correlates of motor expertise comparing athletes and non-athletes using fNIRS (Seidel et al., 2017, 2019). In combination with novel approaches such as multi-channel whole-brain fNIRS, multi-distance fNIRS (Kenville et al., 2017; Seidel et al., 2019) and systemic physiological augmented fNIRS (Herold et al., 2018), these studies provide an important basis for neurodiagnostics of motor expertise and talent (see Figure 1B). Furthermore, a combination of non-invasive brain imaging techniques might help to overcome limitations in spatial and/or temporal resolution. For example, simultaneous EEG and fNIRS recordings (Ludyga et al., 2019) might contribute to shed more light on the question when specific brain networks are active during sport-specific movements and if these networks change during the time course of training.

### Systematic Assessment of Training-Induced Neuroplasticity

Apart from quantifying brain function and structure in athletes, the question remains whether neuroplasticity in athletes is training-induced or an epiphenomenon of genetic predisposition. To address this question, several longitudinal training interventions have been conducted over the past decade (Draganski et al., 2004; Taubert et al., 2010, 2011; Tomassini et al., 2011; Gryga et al., 2012; Zatorre et al., 2012; Sehm et al., 2014). Using MRI, it has been shown that not only motor skill learning over several weeks, but also short-term training can lead to specific structural and functional brain adaptations (Floyer-Lea and Matthews, 2005; Kwon et al., 2012). Interestingly, the individual training success seems to be associated with neuroplasticity in motor-related brain regions. For example, participants with the highest learning success in a whole-body balancing task where those that showed the strongest structural brain adaptations (Taubert et al., 2010). Even more interesting, the individual training success seems to be predictable by the individual brain structure before motor skill learning. For example, Gryga et al. (2012) found that participants with the highest density of GM in the cerebellum, an area that plays an important role in processing complex movement patterns, were those with the greatest training outcome in a sequential pinch force task.

Apart from these exciting insights, however, the key limitation of the aforementioned studies is that MRI assessments did not allow online measurements of functional neuroplasticity during motor skill learning and/or training of motor abilities in athletes. Therefore, fNIRS seems to be particularly suitable to quantify functional neuroplasticity systematically during training processes. Here, it seems to be important to assess the temporal dynamics of training-induced neuroplasticity during sport-specific training in real-world settings and its behavioral relevance. This knowledge in turn might be used to control and/or optimize training success in various sports disciplines. Furthermore, neurodiagnostics in sports could also be used in the field of talent diagnostics. Here, certain particularities in brain function and/or structure of young athletes might be used as a predictor for their potential of becoming an elite athlete, their prerequisites to acquire specific skills or to improve motor performance or even their suitability to a specific sports discipline.

### Performance Enhancement Using Non-invasive Brain Stimulation

Identifying training-induced neuroplasticity is a prerequisite for targeted neuromodulation to augment motor performance and/or sport-specific skills (see Figure 1B). Here, non-invasive brain stimulation methods such as transcranial direct current stimulation (tDCS) are capable of modulating neural processing in specific brain areas and thereby influence motor behavior (Nitsche and Paulus, 2000; Stagg and Nitsche, 2011). While the exact underlying mechanisms of tDCS-induced effects on a cortical and behavioral level remain elusive, there is accumulative evidence that tDCS induces a polarity dependent modulation of the resting membrane potential (Priori et al., 1998; Nitsche and Paulus, 2000). More specifically, anodal tDCS has been shown to increase resting membrane potential while cathodal tDCS decreases it (Nitsche et al., 2003a; Gandiga et al., 2006). This modulation can subsequently lead to either an increase or decrease of neuronal excitability that can outlast the stimulation period by several minutes or even hours (Nitsche and Paulus, 2000, 2001; Lang et al., 2004, 2005; Nitsche et al., 2005).

For example, a single tDCS session has been shown to increase motor performance or skill learning (Nitsche et al., 2003b; Vollmann et al., 2013; Ammann et al., 2016; Kaminski et al., 2016; Jackson et al., 2019; Kumari et al., 2019). tDCS-induced performance enhancement has not only been described for simple motor tasks such as tapping (Saimpont et al., 2016) and reaction time tasks (Nitsche et al., 2003b; Drummond et al., 2017; Hupfeld et al., 2017), but also for complex whole-body tasks such as balancing (Dutta et al., 2014; Kaminski et al., 2016). Moreover, further studies demonstrated that tDCS is capable of increasing endurance performance during cycling (Okano et al., 2015; Vitor-Costa et al., 2015; Angius et al., 2018a; Park et al., 2019) and running (Park et al., 2019) as well as leg muscle power (Tanaka et al., 2009). Tanaka et al. (2009) revealed that a single session of anodal tDCS transiently enhanced maximal leg pinch force by approx. 15% in normal volunteers. Imagine the importance of such performance enhancements via tDCS in competitive sports where even a subtle change in performance decides about winning or losing.

Neuromodulation to augment performance in sports is no science fiction. Several opinion papers and systematic review and meta-analysis articles discussed the feasibility of tDCS as a performance enhancer in athletes (Bolognini et al., 2009; Banissy and Muggleton, 2013; Davis, 2013; Reardon, 2016; Edwards et al., 2017; Angius et al., 2018b; Machado et al., 2019). Interestingly, tDCS is capable of increasing isometric strength (Hazime et al., 2017; Vargas et al., 2018), countermovement jump performance (Lattari et al., 2020) and endurance performance (Okano et al., 2015) even in trained athletes. These findings indicate that tDCS, if suitably applied, might potentially have positive effects on the athlete’s performance. However, the effectiveness and relevance of tDCS in a sport-specific context has to be investigated more thoroughly in future studies. Furthermore, the exact parameters for tDCS applications in sports remain elusive. For example, it needs to be further clarified e.g., when tDCS should be applied to successfully modulate performance in athletes, i.e., before, during or after training sessions? How long and intense should be stimulated? How often should tDCS be applied concerning training and competition? Days, hours or minutes before the competition? How long-lasting are tDCS effects?

Regardless of these open methodological questions, it is by no means clear if tDCS outside highly controlled laboratory settings is at all effective in boosting performance during competition, especially in highly trained athletes. On the one hand, tDCS is known to induce very variable effects on a behavioral level (Bashir et al., 2019). On the other hand, it is important to keep in mind that athletes already show a kind of ceiling effect in their performance which might potentially lead to no detectable tDCS effects or even a decrement in performance. Furthermore, it is necessary to consider the ethical aspects of the use of tDCS to improve sporting performance (Banissy and Muggleton, 2013). This, in turn, will also be a challenge not only for sports authorities to determine where the tDCS application fits into the regulatory framework at the elite level (Edwards et al., 2017). To date, there are no reliable data on possible negative long-term effects of tDCS, especially after repeated and regular use. Hence, it seems to be reasonable to first understand the exact underlying mechanisms and to quantify optimal stimulation parameters to use non-invasive brain stimulation in athletes more effectively.

## Conclusion

In summary, we suggest that neurodiagnostic tools such as MRI, EEG or fNIRS should be implemented in performance diagnostics in sports. Since optimal brain processing is a key factor for efficient motor control and performance, characterizing adaptational brain alterations as a consequence of systematic training might open novel perspectives to augment training success in athletes. Therefore, a desirable goal of future neurodiagnostics is to identify brain networks that contribute to performance improvement in general and, beyond that, brain networks that are particularly responsible for the execution of specific sports disciplines. Furthermore, neurodiagnostics might help to identify youth athletes with the potential of becoming elite athletes. Additionally, neuromodulation might be an alternative way to optimize training outcomes by a selective modulation of performance-relevant brain regions. However, it first has to be shown that tDCS in athletes is at all capable of enhancing motor skill learning and/or motor performance, and if so, that this is performance-relevant and beneficial in specific sports disciplines. Finally, the development and application of neuromodulation in sports must be accompanied by a continuous discussion concerning framework conditions such as ethical aspects, risks, and implementation in the field.

## Author Contributions

All authors contributed to the manuscript, reviewed it, approved the final version content and agree to be accountable for all aspects of the work. All persons designated as authors qualify for authorship, and all those who qualified for authorship are listed.

## Conflict of Interest

The authors declare that the research was conducted in the absence of any commercial or financial relationships that could be construed as a potential conflict of interest.
